# Age and sex and their influence on the anatomy of the abdominal aorta and its branches

**DOI:** 10.1590/1677-5449.200073

**Published:** 2020-12-11

**Authors:** Adenauer Marinho de Oliveira Góes, Flávia Beatriz Araújo de Albuquerque, Fernanda Acatauassú Beckmann, Fernanda Vieira Centeno, Mariseth Carvalho de Andrade, Waldonio de Brito Vieira

**Affiliations:** 1 Centro Universitário do Estado do Pará – CESUPA, Faculdade de Medicina, Belém, PA, Brasil.; 2 Fundação Santa Casa de Misericórdia do Pará – FSCMPA, Departamento de Radiologia, Belém, PA, Brasil.

**Keywords:** aging, remodeling, tomography, anatomy, arteries

## Abstract

**Background:**

It is not clear how patients’ age and sex influence the anatomy of the aorta and its branches.

**Objectives:**

To determine the most frequent anatomical patterns of diameter and angulation of the abdominal aorta and its branches and the influence of patients’ sex and age on these patterns.

**Methods:**

CT scans with intravenous contrast from 157 patients were analyzed. Diameter and angulations of the abdominal aorta and its branches were measured in individuals of both sexes, classified into five age groups: 20 to 30 years, 31 to 40 years, 41 to 50 years, 51 to 60 years, and 61 to 70 years. Eighteen variables were analyzed: 6 arterial origin angles, 9 arterial diameters, rate of diameter enlargement, and patient’s sex and age. RadiAnt 4.2.1 DICOM viewer software was used for measurements.

**Results:**

The total of 157 CT scans were from 69 men and 88 women. There were statistical differences (p <0.05) in the following results: angle of origin and diameter of the superior mesenteric artery; renal artery angle and diameter; diameter of the common iliac arteries, and diameter and rate of diameter enlargement of the aorta in several segments, but not the segment immediately proximal to the celiac trunk.

**Conclusions:**

The diameters of several aorta segments and of its branches (except the left renal artery) increase progressively with age in both sexes and are larger and have a higher rate of diameter enlargement in men than in women in the same age ranges. Between sexes, the angle of origin of the superior mesenteric artery was larger in men, except between 20 and 30 years, and the angle of origin of the left renal artery was larger in women between 51 and 60 years old.

## INTRODUCTION

Anatomic knowledge is indispensable for planning and execution of surgical procedures, whether performed by open or endovascular approaches.^1^^-^4 The anatomy of the cardiovascular system undergoes changes related to age, lifestyle habits, and diseases. Aging causes structural and functional changes, particularly in the major arteries.^5^^-^11 These changes result in increased vascular rigidity due to increased production and deposition of collagen and loss of elastin fibers, primarily in the tunica media of large and medium arteries.^5^^,^^9^^,^^10^^,^^12^^-^14

Vascular rigidity has been known as a risk factor for cardiovascular diseases since the nineteenth century.^9^^,^^14^ However, noninvasive methods that enable the anatomy and physiology of the circulatory system to be studied (blood flow, diameter, angles, and other details), relating them to their clinical repercussions and utilities, have only recently become available.^13^^,^^14^

Studies have already proven that aging affects vascular changes differently in men and women and while there is already evidence of biochemical and functional differences,^6^ much still remains to be investigated in relation to the changes to the arterial anatomy that occur as aging advances and in relation to how patient sex influences these changes.

The objectives of this study were to determine the most frequent anatomic patterns of diameter and angles of the abdominal aorta and its branches and the influence of patients’ sex and age on these patterns.

## METHODS

This is an analytical, descriptive, and retrospective study based on anatomic measurements of abdominal arteries examined using computed tomography (CT). It was approved by the institutional ethics committee (decision number 2.621.934).

The inclusion criteria were: patients of both sexes, aged from 20 to 70 years, who underwent an abdominal CT with intravenous contrast from January 2015 to September 2018. Exclusion criteria were: technical inability to perform the measurements, diseases that change vascular anatomy, such as aneurysms, vascular compression syndromes, and tumors with blood vessel distortions, among others, and anatomic variants, such as accessory/polar renal arteries and anomalous origins of visceral arteries.

No sample size calculation was performed. The sample comprised all examinations provided by a radiology service that partners the university that were conducted within the study period and met inclusion and exclusion criteria.

Examinations were conducted in a GE healthcare 16-channel CT scanner, with a 512 × 512 resolution matrix and slice thickness of 1.25 mm. Data were organized using a standardized protocol developed by the researchers and RadiAnt 4.2.1 DICOM viewer software (Medixant, Poznan, Poland) was used to perform measurements.

**Figure 1:**
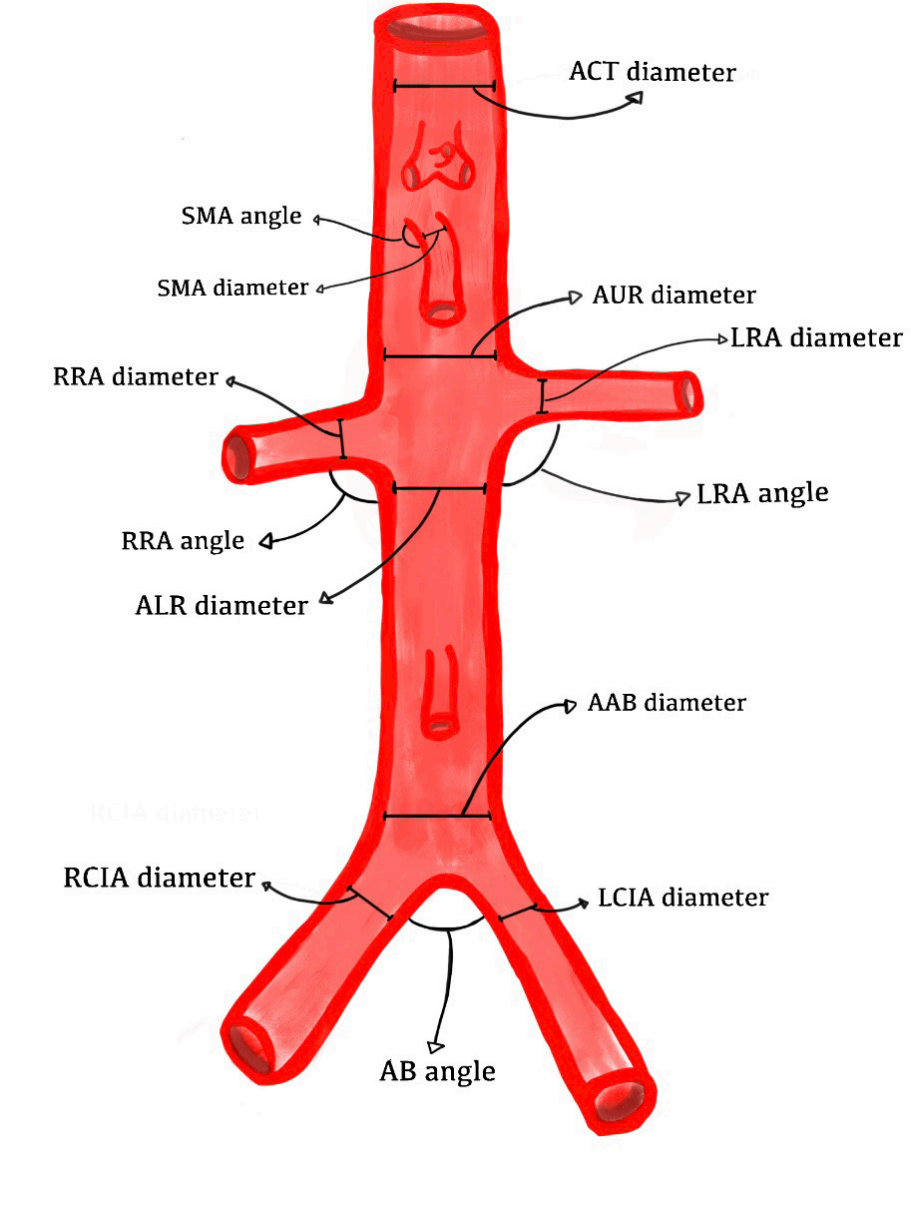
Points at which angles and diameters of the abdominal aorta and its branches were measured. SMA: superior mesenteric artery; RRA: right renal artery; LRA: left renal artery; AB: aortoiliac bifurcation; ACT: aorta proximal of the celiac trunk; AUR: aorta proximal of the upper renal artery; ALR: aorta distal of the lower renal artery; AAB: aorta proximal of the aortoiliac bifurcation; RCIA: right common iliac artery; LCIA: left common iliac artery [[Q1: Q1]].

The following variables were analyzed by patient sex and age groups (20 to 30 years, 31 to 40 years, 41 to 50 years, 51 to 60 years, and 61 to 70 years): diameter, angle of origin, and rate of diameter enlargement of the superior mesenteric arteries (SMA), right renal arteries (RRA) and left renal arteries (LRA), the aortoiliac bifurcation (AB) angle, and the diameter and dilation index of the aorta in 4 different segments, specifically, proximal of the celiac trunk (ACT), proximal of the upper renal artery (AUR), distal of the lower renal artery (ALR), and proximal of the aortoiliac bifurcation (AAB), and the diameter and dilation index of the right common iliac artery (RCIA) and left common iliac artery (LCIA), as illustrated in Figure 1.

For statistical analysis, the Shapiro-Wilk test was used to confirm normality of values; Student’s *t* test was used for comparisons by sex, and analysis of variance (ANOVA) was used for comparisons between age groups. The dilation index for each sex was calculated by subtracting the mean diameter found in the oldest age group (61 to 70 years) from that found in the youngest age group (20 to 30 years), dividing the result by the mean diameter in the youngest age group and then multiplying by 100; as in the following formula: DM1-DM2/DM2 x 100 (where DM1 is the mean diameter in the oldest age group and DM2 is the mean diameter in the youngest age group). BioEstat® 5.4 (Ayres, Belém) software was used and the significance level adopted was α = 0.05 or 5%.

## RESULTS

A total of 198 CTs were analyzed. After application of exclusion criteria, 41 were rejected. The final sample therefore comprised 157 CTs, 69 from men and 88 from women, distributed across the following age groups: 20 to 30 years (20 patients); 31 to 40 years (24 patients); 41 to 50 years (35 patients); 51 to 60 years (42 patients); and 61 to 70 years (36 patients).

### Superior mesenteric artery

**Figure 2:**
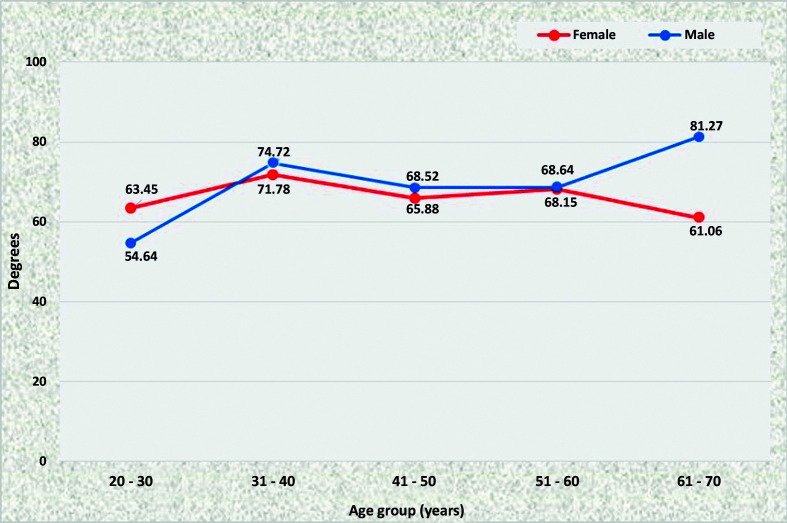
Mean superior mesenteric artery angles for both sexes in the preestablished age groups.

The angle of origin of the SMA was statistically similar for men and women in all age groups, except from 61 to 70 years, in which male patients had a mean angle of 81.27° while female patients had a mean angle of 61.06°. Among female patients, this angle also did not vary significantly with increasing age, whereas in older men the SMA tended to emerge at an angle that was around 26° larger than in younger men (81.27° in the seventh decade of life vs. 54.64° in the third decade) (Figure 2).

**Figure 3:**
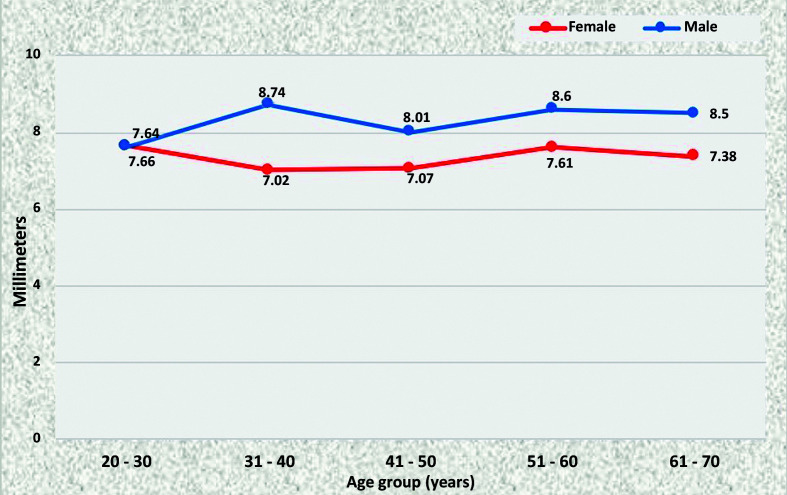
Mean diameters of the superior mesenteric artery for both sexes in the preestablished age groups.

The SMA diameter was similar among young men and women (7.66 mm for women aged 20 to 30 years and 7.64 mm for males in the same age group), but in older age groups this diameter was statistically larger among men (Figure 3).

### Renal arteries

**Figure 4:**
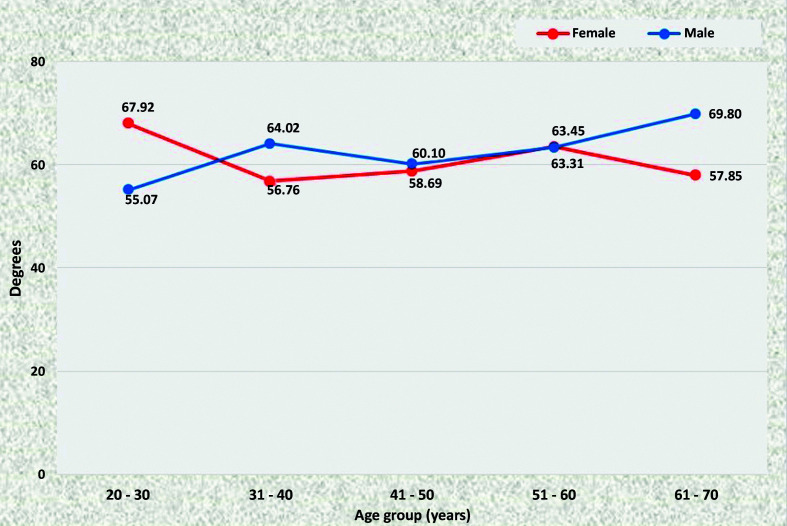
Mean right renal artery angles for both sexes in the preestablished age groups.

The right renal artery (RRA) had a larger angle (mean = 67.92°) in young female patients (20 to 30 years) than in male patients in the same age group (mean = 55.07°) (p = 0.0335). The inverse relationship was observed in the oldest age group (61 to 70), in which men had a mean angle of 69.80° and women had a mean angle of 57.85° (p = 0.0140). There was no statistical difference in this angle as age increased in either sex (Figure 4).

**Figure 5:**
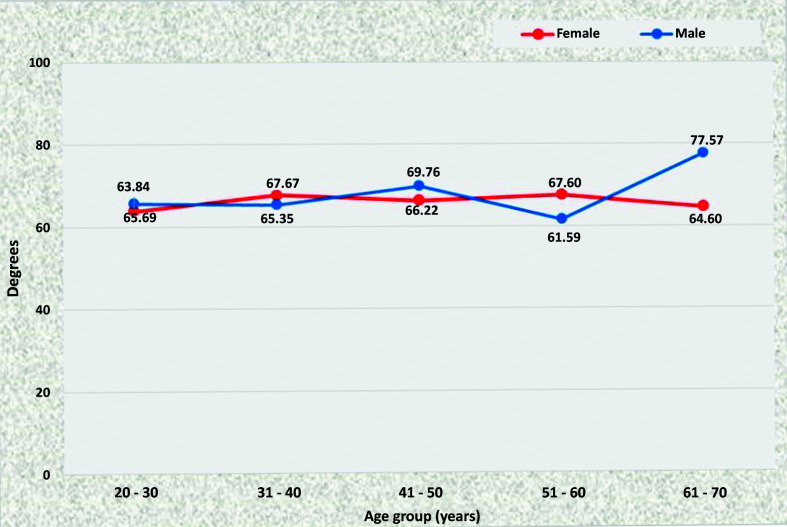
Mean left renal artery angles for both sexes in the preestablished age groups.

On the left, the renal artery (LRA) also did not exhibit a statistically significant difference in angle as age increased when individuals of the same sex were compared and, in common with the RRA, the angle of origin was larger among older men (61 to 70 years) than among women in the same age group (means of 77.57° and 64.60° with p = 0.0084) (Figure 5).

**Figure 6:**
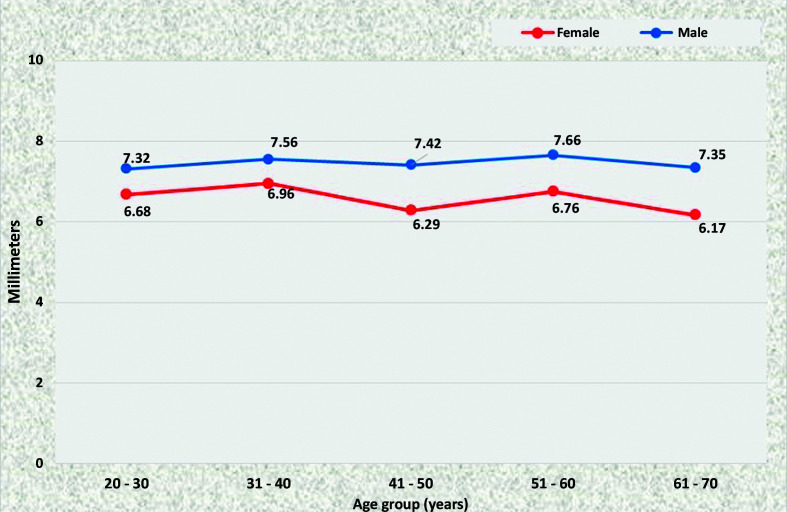
Mean diameters of the right renal artery for both sexes in the preestablished age groups.

**Figure 7:**
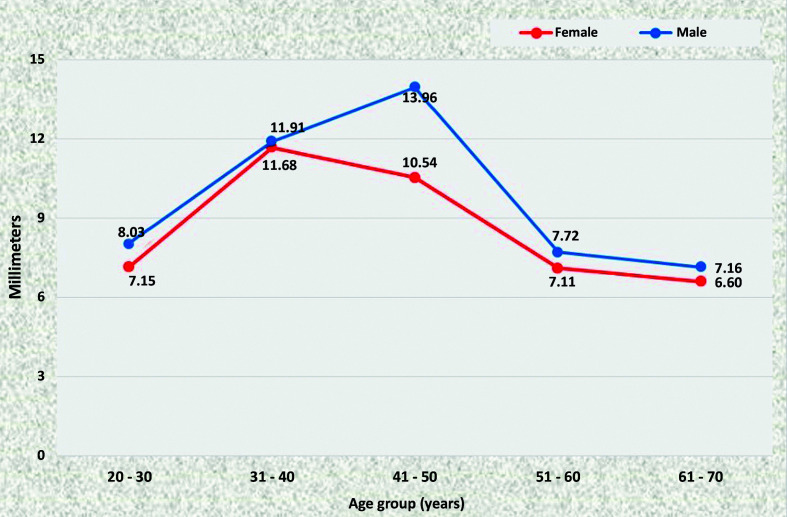
Mean diameters of the left renal artery for both sexes in the preestablished age groups.

The diameters of the renal arteries were larger on the right; men aged from 41 to 70 years had larger RRA diameters than women in the same age range, whereas the LRA was statistically of larger caliber in men for the 20 to 30 years age group only (p = 0.0354). The diameters of the renal arteries are shown in Figures 6 and 7.

### Common iliac arteries

**Figure 8:**
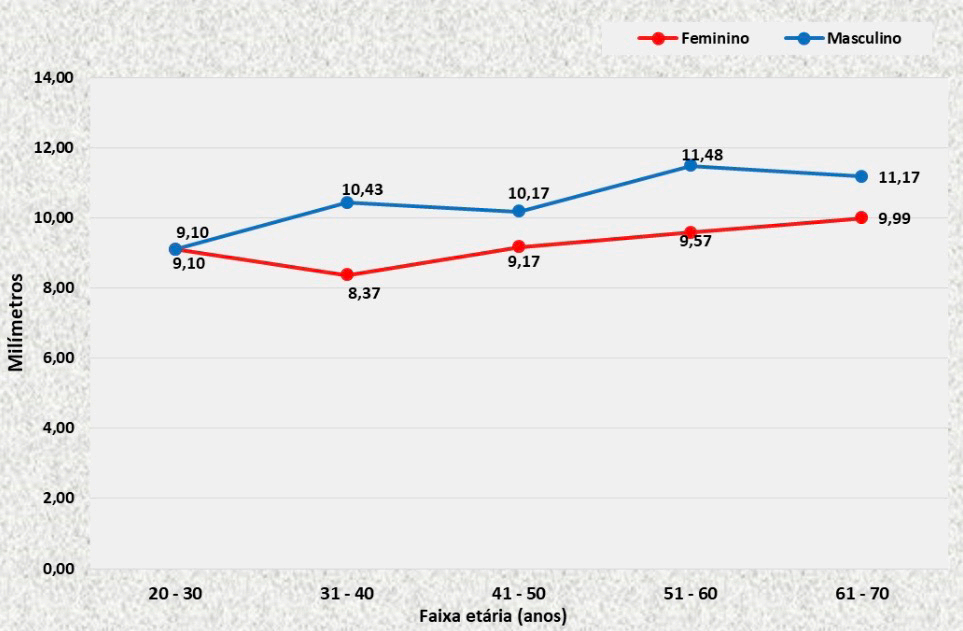
Mean diameters of the right common iliac artery for both sexes in the preestablished age groups.

**Figure 9:**
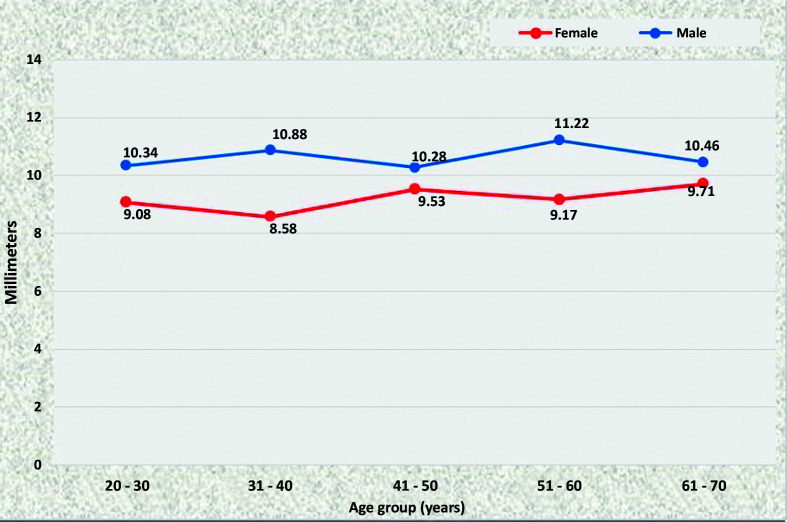
Mean diameters of the left common iliac artery for both sexes in the preestablished age groups.

The common iliac arteries tend to have larger caliber in men than in women. On the right side, this was the case in the majority of age groups, with the exception of the youngest patients, among whom the mean diameter was the same in both sexes (Figure 8). On the left side, the common iliac artery was statistically of larger caliber in men aged 20 to 30 years, 31 to 40 years, and 51 to 60 years than in women in the same age groups (Figure 9).

The iliac arteries progressively increased in diameter as age increased in both sexes; but the increase in diameters was only statistically significant in men, specifically on the right (p = 0.0227).

### Angle of the aortic bifurcation

**Figure 10:**
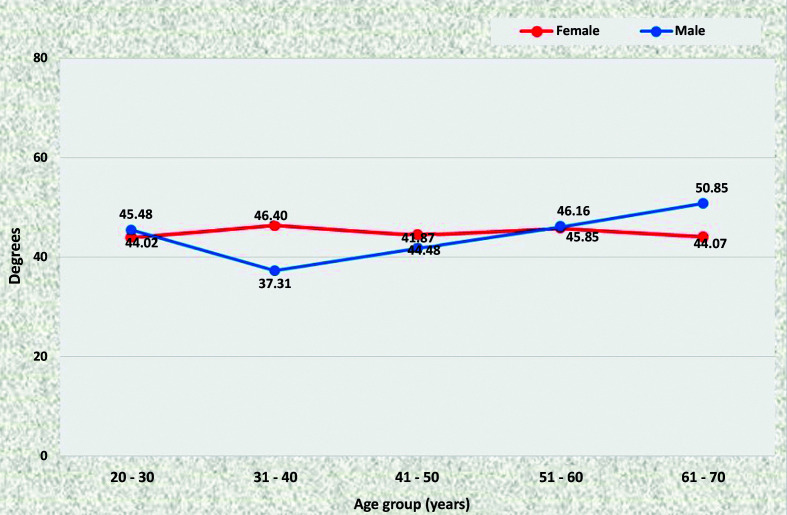
Mean aortoiliac bifurcation angle for both sexes in the preestablished age groups.

Among women, the mean angle remained in the region of 44° in all age groups, whereas in men it varied from 45.48° from 20 to 30 years to 50.85° from 61 to 70 years, although

without statistical difference. There was only a statistically significant difference between patients of different sexes in the 31 to 40 years age group, in which the angle was larger in women (46.40°) than in men (37.31°) (p = 0.0187) (Figure 10).

### Mean diameters of the abdominal aorta

**Figure 11:**
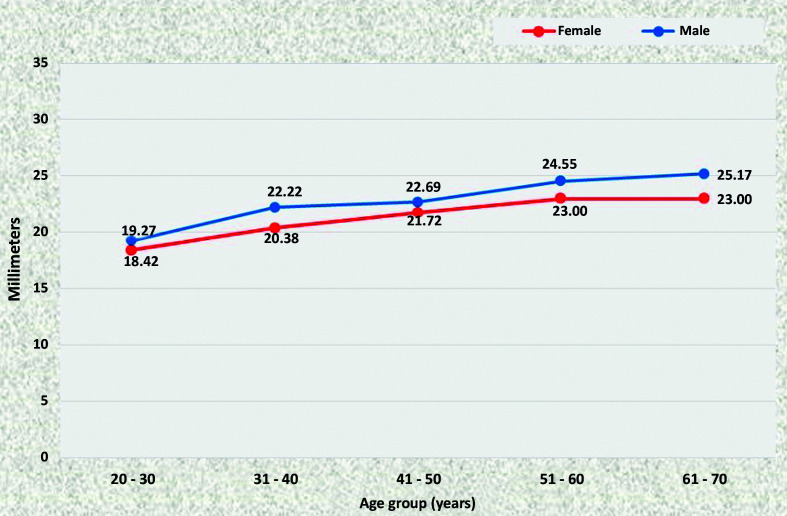
Mean diameters of the aorta proximal of the celiac trunk for both sexes in the preestablished age groups.

**Figure 12:**
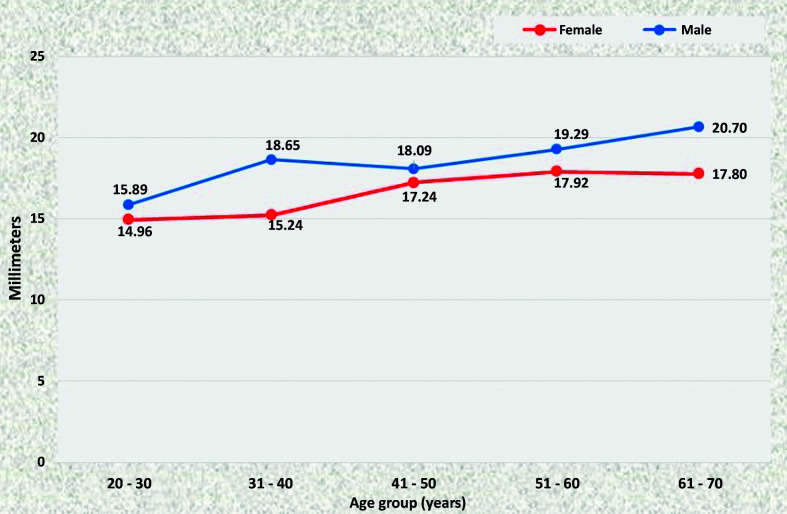
Mean diameters of the aorta distal of the lower renal artery for both sexes in the preestablished age groups.

**Figure 13:**
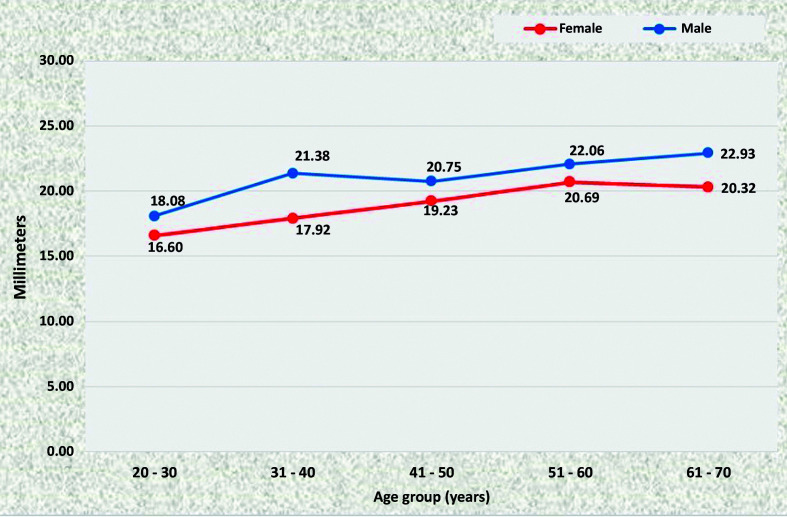
Mean diameters of the aorta proximal of the upper renal artery for both sexes in the preestablished age groups.

**Figure 14:**
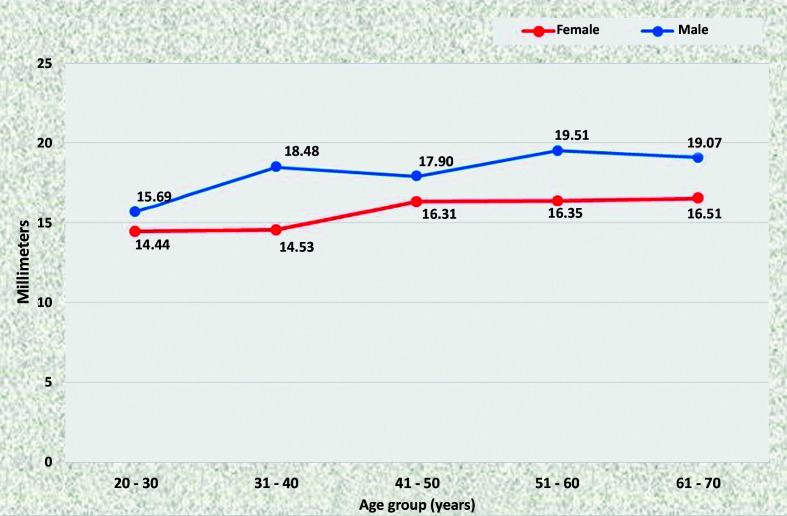
Mean diameters of the aorta proximal of the aortoiliac bifurcation for both sexes in the preestablished age groups.

At all points, statistically significant increases in the diameter of the aorta were observed as age increased, in both men and women. There was a trend for all diameters to be larger in men than in women. In the ACT (Figure 11) and ALR segments (Figure 12), differences were statistically significant in the age groups 31 to 40, 51 to 60, and 61 to 70, whereas in the AUR (Figure 13) and AAB segments (Figure 14) diameters were statistically larger in all age groups.

### Rate of diameter enlargement

**Figure 15:**
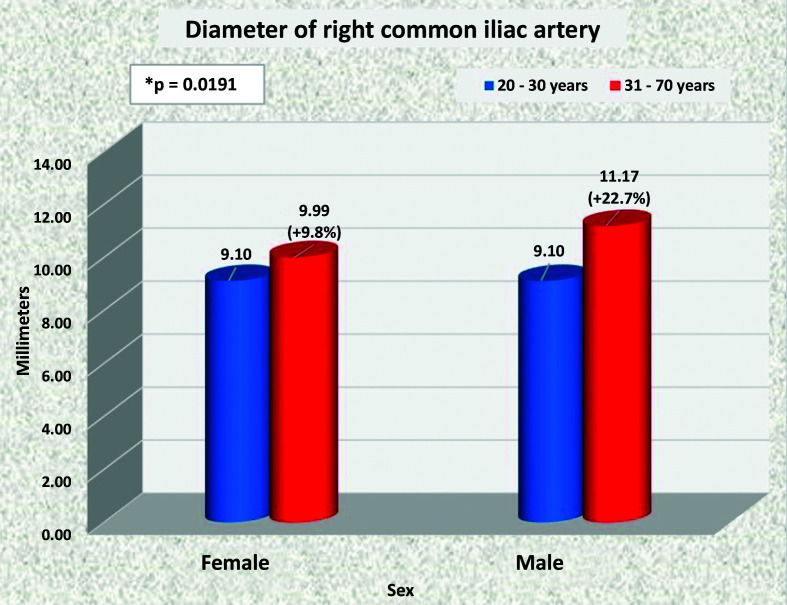
Comparison of dilation indexes for men and women in the 20-30 and 61-70 years age groups. *: p<0.05.

The rate of diameter enlargement was significantly higher in men in the following segments: SMA (p = 0.0023); RRA (p = 0.0003), and RCIA (p = 0.0191) (Figure 15).

**Figure 16:**
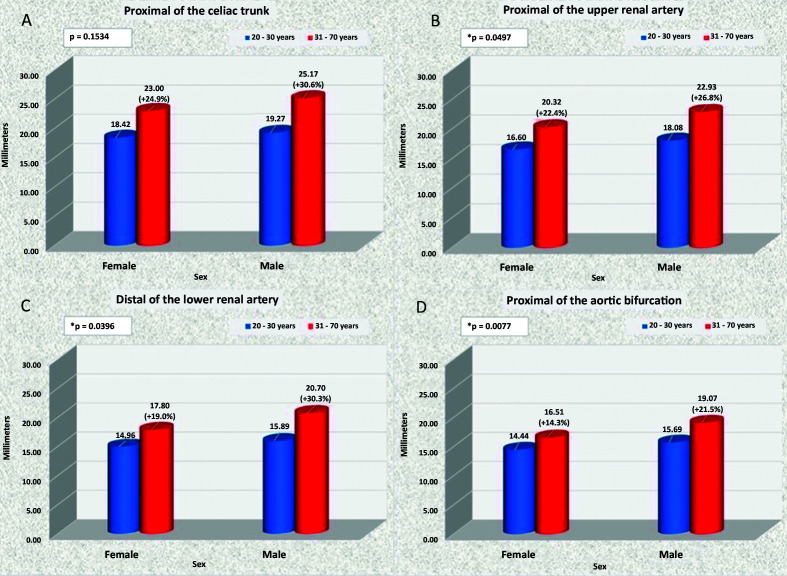
Comparison of dilation indexes for abdominal aorta segments in men and women in the 20-30 and 61-70 years age groups. In (A), in the segment proximal of the celiac trunk; in (B), proximal of the upper renal artery; in (C), distal of the lower renal artery; and in (D), proximal of the aortic bifurcation. *: p<0.05.

Moreover, when patients in the oldest and youngest age groups were compared, the rates of diameter enlargement for the aortic segments analyzed were statistically higher in men than in women (p < 0.05), with the exception of the segment immediately proximal of the celiac trunk (Figure 16).

## DISCUSSION

Aging is a dynamic, progressive, and irreversible process, intimately linked to biological, psychological, and social factors.^5^ Its effects are not only dependent on age, but also on sex, lifestyle, comorbidities, socioeconomic factors, and constitutional influences. This is why the elderly population is not uniform.^5^^,^^10^^,^^15^^-^17 Surgeons should strive to acquire the most detailed knowledge possible of the structures involved in each procedure performed, because this has positive impacts on the quality of the technique employed and on avoidance of iatrogenic injuries.

The aorta and other large caliber arteries, particularly those with a more developed elastic layer, become more dilated, stretched, and tortuous as patients age.^13^^,^^18^^,^^19^ Understanding of the changes arterial anatomy undergoes as it ages can be useful when planning invasive procedures, for example, when choosing approaches, the curvature of catheters, and the diameters of angioplasty balloons and stents in endovascular procedures, and, in the future, may also be of use in the manufacture of endovascular devices with diameters and curvatures that fit patients better on the basis of their sex and age group.

**Figure 17:**
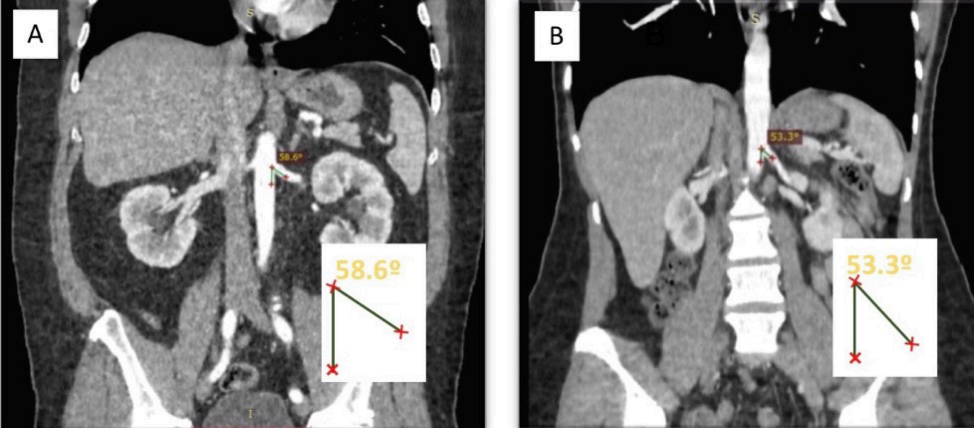
Abdominal computed tomography with contrast in coronal view, comparing measurements of the angle of origin of the left renal artery (LRA) in patients of both sexes aged 61 to 70 years. (A) angle of origin of the LRA in a man; (B) angle of origin of the LRA in a woman.

Obviously, when available, planning of an intervention should take into consideration anatomic information provided by noninvasive examinations; but the results of this study should nevertheless help to prevent certain difficulties. For example, when attempting selective catheterization of the LRA in a 68-year-old patient using a right femoral artery puncture for access, based on the differences in angles observed in the present study, it is probable that a female patient would present greater difficulty than a male in the same age group (Figure 17).

It is of interest to note that the study detected a greater tendency in men to dilation of arteries as age advances, such as the abdominal aorta and RCIA, as has been suggested before in other studies.^17^^-^22

The SMA also exhibited greater changes in angle and diameter in men. The lesser predisposition towards changes in arterial anatomy among women may be related to the role played by estrogen,^6^^,^^9^^,^^22^ which increases the bioavailability of nitric oxide (NO) without increasing expression and/or activity of endothelial nitric oxide-synthase (eNOS), which has antioxidant properties that may induce or accelerate vascular aging.^6^^,^^8^^,^^9^ Nitric oxide bioavailability is essential for normal endothelial function and it is known that advanced age leads to compromised endothelial NO production and increased NO inactivation by superoxide, contributing to age-related endothelial dysfunction.^6^^,^^10^^,^^16^^,^^23^^,^^24^

The most notable characteristics of vascular aging described in the literature are the mechanical and structural changes to vessel walls, including arterial dilation and thickening, primarily seen in the abdominal aorta and highlighted in many publications.^5^^,^^12^^,^^15^^,^^22^^,^^25^ Arterial changes over the course of life are also influenced by modifiable cardiovascular risk factors, such as hypertension, obesity, smoking, and lifestyle, and also by unmodifiable factors, such as genetics, age, and family history.^15^^,^^16^^,^^21^

It is known that aortic rigidity increases after 50 years of age even in healthy patients^7^^,^^26^ and post-mortem studies show that thickening of the aorta wall during aging occurs through increased tunica intima thickness, even in populations with low incidence of atherosclerosis.^7^^,^^8^^,^^10^^,^^15^^,^^17^ Anatomically, these changes do not only manifest as increased diameter, but also as aortic elongation, and, from a physiological point of view, arterial thickening, which is related to pulse wave velocity, and has been shown to be an independent risk factor for cardiovascular events.^5^^,^^9^^,^^13^^,^^14^

Although we did not investigate thickening, the changes to arterial diameter detected in this study corroborate findings in the literature. However, in a review of the literature, we did not find any studies that had correlated the angles of emergence of the branches of the abdominal aorta with patient sex and age.

**Figure 18:**
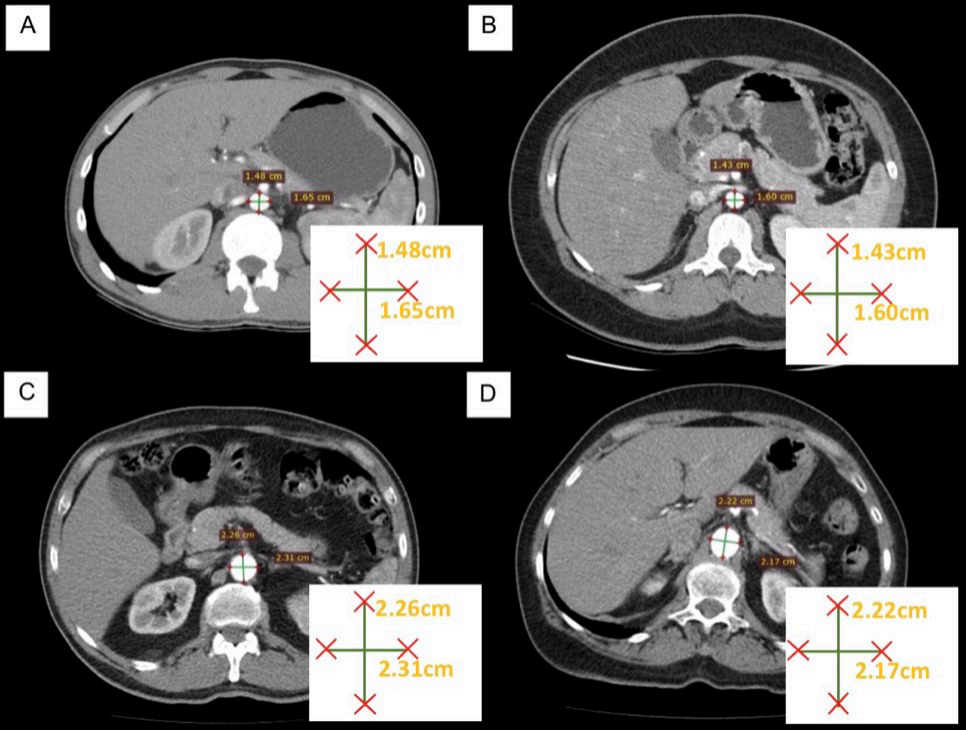
Abdominal computed tomography with contrast in axial view, comparing measurements of diameters of the abdominal aorta proximal of the upper renal artery in patients of both sexes and different age groups. (A) male patient in the 20-30 years age group; (B) female patient in the 20-30 years age group; (C) male patient in the 60-70 years age group; (D) female patient in the 60-70 years age group.

Unfortunately, the retrospective nature of this study only enabled access to sex and age group of the patients examined, which meant that it was only possible to confirm that the diameter of the aorta was larger in men and that it increased progressively with age in both sexes (Figure 18). Several published articles have shown that the diameters of the aorta are larger in men than in women,^12^^,^^18^^-^20^,^^22^^,^^25^^,^^26^ but did not determine that the dilation index of these diameters is greater among men than among women in all age groups, as was detected in the present study.

Just as occurred with the aorta, the RCIA and LCIA also exhibited larger mean diameters in men than in women and the RCIA exhibited a significant increase in caliber as age increased.

The literature on aneurysms of the iliac arteries is scant, but there is evidence to suggest common iliac arteries without aneurysms have larger caliber on the right than on the left and that when aneurysms do occur they are both more frequent and tend to have larger diameter on the right,^21^^,^^27^^-^29 coinciding with the findings in our sample. This fact may be related to greater occurrence of aneurysms in the aorta (4 male cases:1 female case)^30^ and iliac arteries (5 male cases:1 female case),^27^ since the Laplace law states that the larger the radius of a vessel, the greater the tension exerted on its wall.^31^ In other words, since men have larger caliber vessels, they may have an anatomic predisposition to develop aneurysms in these arteries.

Obviously, this observation does not alone explain the increased incidence of aneurysms in segments of smaller diameter, since it is known that aneurysms of the abdominal segment of the aorta are around four times more frequent than those in the thoracic aorta, which has a larger diameter.^13^^,^^28^ The origins of a complex disease such as an aneurysm are undoubtedly multifactorial. It is known, for example, that there are considerable histological changes in the aorta over the course of life, since the aortas of young people have thick elastin fibers that are concentric and uniform, whereas in the elderly they are thinner and more fragmented, occupying a smaller volume in the tunica media of the artery.^5^^,^^7^^,^^10^^,^^14^^,^^32^^,^^33^

The ideal method for evaluating changes to arterial anatomy associated with age in patients of both sexes would be to follow a cohort of patients using imaging exams over the course of their lives. However, such a study design would have to overcome enormous obstacles to its execution. Other limitations of the present study include a relatively small number of CTs and the absence of information on patients’ comorbidities and lifestyle habits, such as smoking, which could predispose to changes to arterial anatomy.

## CONCLUSIONS

The diameter of many different segments of the aorta increase progressively as age increases in patients of both sexes and diameters are larger in men than in women among patients in the same age groups. With the single exception of the LRA, the diameters of all of the branches of the aorta were larger in men than in women in all age groups. The angle of origin of the superior mesenteric artery was larger in men than in women in all age groups except for 20 to 30 years. The angle of origin of the left renal artery was larger in women than in men in the 51 to 60 years age group. With the exception of the aortic segment proximal of the celiac trunk, the dilation indexes for the diameters of all other segments of the abdominal aorta were higher in men than in women.
