# Supplementary material for: Age and sex and their influence on the anatomy of the abdominal aorta and its branches
Source: J Vasc Bras. 2020 Dec 11;19:e20200073. doi: 10.1590/1677-5449.200073 (PMC8218012; doi:10.1590/1677-5449.200073)
Supplement: Translation

# Translation: Sexo e idade e sua influência sobre a anatomia da aorta abdominal e seus ramos

**Authors:** Adenauer Marinho de Oliveira Góes, Flávia Beatriz Araújo de Albuquerque, Fernanda Acatauassú Beckmann, Fernanda Vieira Centeno, Mariseth Carvalho de Andrade, Waldonio de Brito Vieira

**Affiliations:** 1 Centro Universitário do Estado do Pará – CESUPA, Faculdade de Medicina, Belém, PA, Brasil.; 2 Fundação Santa Casa de Misericórdia do Pará – FSCMPA, Departamento de Radiologia, Belém, PA, Brasil.

**Keywords:** envelhecimento, remodelação, tomografia, anatomia, artérias

## Abstract

**Contexto:**

Não se sabe ao certo como a idade e o sexo do paciente influenciam na anatomia da aorta abdominal e de seus ramos.

**Objetivos:**

Determinar os padrões anatômicos (diâmetro e angulações) mais frequentes da aorta abdominal e de seus ramos e a influência do sexo e da idade dos pacientes sobre esses padrões.

**Métodos:**

Foram avaliadas tomografias computadorizadas de abdome com contraste endovenoso de 157 pacientes. Foram aferidos calibre e angulação de artérias abdominais em indivíduos de ambos os sexos, agrupados em cinco faixas etárias: 20 a 30 anos, 31 a 40 anos, 41 a 50 anos, 51 a 60 anos e 61 a 70 anos. Foram analisadas 18 variáveis: seis ângulos de emergências arteriais, nove diâmetros arteriais, taxas de dilatação, sexo e faixa etária. Para a obtenção das medidas, utilizou-se o programa de computador RadiAnt 4.2.1 DICOM *viewer* (Medixant, Poznan, Polônia).

**Resultados:**

Entre as 157 tomografias, 69 eram de homens e 88, de mulheres. Apresentaram diferença estatística (p < 0,05): ângulo de origem e diâmetro da artéria mesentérica superior; ângulo e diâmetro das artérias renais; diâmetro das artérias ilíacas comuns; diâmetro e taxa de dilatação em diversos segmentos da aorta, exceto na porção proximal ao tronco celíaco.

**Conclusões:**

Os diâmetros da aorta (em diversos segmentos) e de seus ramos (exceto da artéria renal esquerda) aumentam progressivamente com o passar da idade em ambos os sexos e são maiores e possuem taxa de dilatação mais elevada em homens do que em mulheres da mesma faixa etária. Entre os sexos, o ângulo de emergência da artéria mesentérica superior foi maior em homens, exceto entre 20 e 30 anos; o ângulo de origem da artéria renal esquerda foi maior em mulheres entre 51 e 60 anos.

## INTRODUÇÃO

O conhecimento anatômico é indispensável para o planejamento e a execução de procedimentos cirúrgicos por via aberta e endovascular^1^^-^4. A anatomia do aparelho cardiovascular sofre alterações relacionadas a idade, hábitos de vida e doenças. O envelhecimento provoca mudanças estruturais e funcionais, principalmente nas grandes artérias^5^^-^11. As alterações resultam em um aumento da rigidez vascular devido à maior produção e deposição de colágeno e perda de fibras de elastina, principalmente na camada média de grandes e médias artérias^5^^,^^9^^,^^10^^,^^12^^-^14.

A rigidez vascular é conhecida como um fator de risco para doenças cardiovasculares desde o século XIX^9^^,^^14^, porém, métodos não invasivos que permitem o estudo da anatomia e fisiologia do sistema circulatório (fluxo sanguíneo, diâmetro, angulações, entre outros), relacionando-as às suas utilidades e repercussões clínicas, foram disponibilizados apenas recentemente^13^^,^^14^.

Estudos já comprovaram que o envelhecimento afeta de modo distinto as alterações vasculares em homens e mulheres, e, embora já haja evidências de diferenças bioquímicas e funcionais^6^, ainda há muito a ser pesquisado sobre as alterações da anatomia arterial que ocorrem com o avançar da idade e sobre como o sexo do paciente influencia essas modificações.

Os objetivos da pesquisa foram determinar os padrões anatômicos de diâmetro e angulações mais frequentes da aorta abdominal e de seus ramos e a influência do sexo e da idade dos pacientes sobre esses padrões.

## MÉTODOS

Estudo analítico, descritivo e retrospectivo baseado em medidas anatômicas de artérias abdominais avaliadas por tomografias computadorizadas (TC), aprovado pelo comitê de ética institucional (parecer 2.621.934).

Os critérios de inclusão foram: pacientes de ambos os sexos, com idade entre 20 e 70 anos, submetidos à TC abdominal com contraste endovenoso (EV), no período de janeiro de 2015 a setembro de 2018. Os critérios de exclusão foram: impossibilidade técnica para realização de medidas e doenças que alteram a anatomia vascular, como aneurismas, síndromes de compressão vascular e tumores com distorção de vasos sanguíneos, entre outras, e variações anatômicas, como artérias renais acessórias/polares e origem anômala de artérias viscerais.

Não foi realizado cálculo de tamanho amostral. A amostra foi obtida a partir de todos os exames disponibilizados por um serviço de radiologia parceiro da instituição de ensino, correspondentes ao período do estudo após a aplicação dos critérios de inclusão e exclusão.

Os exames foram realizados em tomógrafo GE *healthcare* de 16 canais, com matriz de resolução de 512 × 512 e espessura de corte de 1,25 mm. Os dados foram organizados por meio de protocolo padronizado, elaborado pelos pesquisadores, e o programa de computador RadiAnt 4.2.1 DICOM *viewer* (Medixant, Poznan, Polônia) foi usado para as medidas.

As seguintes variáveis foram analisadas de acordo com o sexo e as faixas etárias (20 a 30 anos, 31 a 40 anos, 41 a 50 anos, 51 a 60 anos e 61 a 70 anos) dos pacientes submetidos à TC: diâmetro, ângulo de emergência e taxa de dilatação das artérias mesentérica superior (AMS), renais direita (ARD) e esquerda (ARE), ângulo da bifurcação aorto-ilíaca (BA), diâmetro e taxa de dilatação da aorta em 4 segmentos, sendo proximal ao tronco celíaco (ATC), proximal à artéria renal mais alta (ARA), distal à artéria renal mais baixa (ARB) e proximal à bifurcação aorto-ilíaca (ABI) e das artérias ilíacas comuns direita (AICD) e esquerda (AICE), como é possível observar na Figura 1.

**Figura 1:** Pontos de aferição dos ângulos e diâmetros da aorta abdominal e seus ramos. AMS: artéria mesentérica superior; ARD: artéria renal direita; ARE: artéria renal esquerda; BA: bifurcação aorto-ilíaca; ATC: aorta proximal ao tronco celíaco; ARA: aorta proximal à artéria renal mais alta; ARB: aorta distal à artéria renal mais baixa; ABI: aorta proximal à bifurcação aorto-ilíaca; AICD: artéria ilíaca comum direita; AICE: artéria ilíaca comum esquerda.

Na estatística analítica, o teste de Shapiro-Wilk foi utilizado para comprovação da normalidade dos valores; utilizou-se o teste *t* de Student para comparação entre os sexos e análise de variância (ANOVA) como critério para comparação entre as faixas etárias. A taxa de dilatação em cada sexo foi calculada subtraindo-se o diâmetro médio encontrado na faixa mais avançada (61 a 70 anos) daquele encontrado na faixa etária etária mais nova (20 a 30 anos), dividindo-se o resultado pelo diâmetro médio da faixa etária mais jovem e multiplicando por 100; conforme a fórmula: DM1-DM2/DM2 x 100 (DM1: diâmetro médio na faixa etária mais avançada; DM2: diâmetro médio na faixa etária mais jovem). Foi utilizado o *software* BioEstat® 5.4 (Ayres, Belém) e adotado o nível de significância α = 0,05 ou 5%.

## RESULTADOS

Foram avaliadas 198 TCs. Após submetidas aos critérios, 41 foram excluídas. Entre as 157 TCs componentes da amostra, 69 eram de homens e 88 de mulheres, distribuídas nas seguintes faixas etárias: 20 a 30 anos (20 pacientes); 31 a 40 anos (24 pacientes); 41 a 50 anos (35 pacientes); 51 a 60 anos (42 pacientes); e 61 a 70 anos (36 pacientes).

### Artéria mesentérica superior

O ângulo de emergência da AMS foi estatisticamente semelhante entre homens e mulheres em todas as faixas etárias, exceto entre 61 a 70 anos, quando em pacientes do sexo masculino esse ângulo foi em média de 81,27°; enquanto, em mulheres, a angulação média foi de 61,06°. Em pacientes do sexo feminino, esse ângulo também não demonstrou variação significativa com o passar da idade, ao passo que em homens mais velhos a AMS tende a emergir em um ângulo cerca de 26° maior do que em homens mais jovens (81,27° na sétima década de vida *versus* 54,64° na terceira) (Figura 2).

**Figura 2:** Ângulos médios da artéria mesentérica superior em ambos os sexos e nas faixas etárias preestabelecidas.

O diâmetro da AMS foi semelhante entre homens e mulheres jovens (7,66 mm entre 20 a 30 anos para o sexo feminino e 7,64 mm para o masculino), porém, em faixas etárias mais avançadas, o diâmetro se tornou estatisticamente maior em homens (Figura 3).

**Figura 3:** Diâmetros médios da artéria mesentérica superior em ambos os sexos e nas faixas etárias preestabelecidas.

### Artérias renais

A artéria renal direita (ARD), em pacientes mais jovens (20 a 30 anos), apresentou angulação maior em mulheres (média = 67,92°) do que em homens (média = 55,07°) (p = 0,0335). Uma relação inversa ocorreu na faixa etária mais avançada (61 a 70), já que em homens o ângulo médio foi de 69,80° e em mulheres, de 57,85° (p = 0,0140). Em nenhum dos sexos houve diferença estatística dessa angulação com o avançar da idade (Figura 4).

**Figura 4:** Ângulos médios da artéria renal direita em ambos os sexos e nas faixas etárias preestabelecidas.

À esquerda, a artéria renal (ARE) também não demonstrou modificação estatisticamente significante da angulação com o avançar da idade quando avaliados indivíduos do mesmo sexo e, assim como ocorreu na ARD, apresentou maior angulação da origem em homens mais velhos (61 a 70 anos) do que em mulheres da mesma faixa etária (médias de 77,57° e 64,60° com p = 0,0084) (Figura 5).

**Figura 5:** Ângulos médios da artéria renal esquerda em ambos os sexos e nas faixas etárias preestabelecidas.

Os diâmetros das artérias renais foram maiores à direita; homens com idade entre 41 e 70 anos apresentaram maior diâmetro da ARD do que mulheres da mesma faixa etária, enquanto a ARE foi estatisticamente mais calibrosa em homens apenas na faixa etária de 20 a 30 anos (p = 0,0354). Diâmetros das artérias renais encontram-se nas Figuras 6 e 7.

**Figura 6:** Diâmetros médios da artéria renal direita em ambos os sexos e nas faixas etárias preestabelecidas.

**Figura 7:** Diâmetros médios da artéria renal esquerda em ambos os sexos e nas faixas etárias preestabelecidas.

### Artérias ilíacas comuns

As artérias ilíacas comuns tendem a ser mais calibrosas em homens do que em mulheres. No lado direito, isso aconteceu na maior parte das faixas etárias, exceto entre os pacientes mais jovens, nos quais o diâmetro médio foi o mesmo em ambos os sexos (Figura 8). No lado esquerdo, a ilíaca comum foi estatisticamente mais calibrosa em homens entre 20 a 30 anos, 31 a 40 anos e 51 a 60 anos do que em mulheres da mesma faixa etária (Figura 9).

**Figura 8:** Diâmetros médios da artéria ilíaca comum direita em ambos os sexos e nas faixas etárias preestabelecidas.

**Figura 9:** Diâmetros médios da artéria ilíaca comum esquerda em ambos os sexos e nas faixas etárias preestabelecidas.

As artérias ilíacas apresentaram aumento progressivo do diâmetro com o passar da idade em ambos os sexos; porém, apenas em homens houve aumento estatisticamente significante dos diâmetros, especificamente à direita (p = 0,0227).

### Ângulo de bifurcação da aorta

Nas mulheres, a média se manteve ao redor de 44° em todas as faixas etárias, enquanto nos homens, embora sem diferença estatística, variou de 45,48° entre 20 a 30 anos a 50,85° entre 61 a 70 anos. Entre pacientes de ambos os sexos, houve diferença estatisticamente significativa apenas na faixa etária de 31 a 40 anos, na qual o ângulo apresentou-se maior em mulheres (46,40°) do que em homens (37,31°) (p = 0,0187) (Figura 10).

**Figura 10:** Ângulos médios da bifurcação aorto-ilíaca em ambos os sexos e nas faixas etárias preestabelecidas.

### Diâmetros médios da aorta abdominal

Em todos os pontos, foram observados aumentos estatisticamente significantes do diâmetro aórtico com o passar da idade, tanto em homens quanto em mulheres. Os diâmetros tendem a ser sempre maiores em homens do que em mulheres. Nas topografias ATC (Figura 11) e ARB (Figura 12), houve significância estatística nas faixas etárias 31 a 40, 51 a 60 e 61 a 70, enquanto nas topografias ARA (Figura 13) e ABI (Figura 14) o diâmetro foi estatisticamente maior em todas as faixas etárias.

**Figura 11:** Diâmetros médios da aorta proximal ao tronco celíaco em ambos os sexos e nas diferentes faixas etárias preestabelecidas.

**Figura 12:** Diâmetros médios da aorta distal à artéria renal mais baixa em ambos os sexos e nas faixas etárias preestabelecidas.

**Figura 13:** Diâmetros médios da aorta proximal à artéria renal mais alta em ambos os sexos e nas faixas etárias preestabelecidas.

**Figura 14:** Diâmetros médios da aorta proximal à bifurcação aorto-ilíaca em ambos os sexos e nas faixas etárias preestabelecidas.

### Taxas de dilatação

Para as seguintes topografias analisadas, a taxa de dilatação foi significativamente maior em homens: AMS (p = 0,0023); ARD (p = 0,0003) e AICD (p = 0,0191) (Figura 15).

**Figura 15:** Comparação da taxa de dilatação entre homens e mulheres nas faixas etárias de 20-30 e 61-70 anos. *: p<0,05.

Além disso, quando comparados pacientes nos extremos de idade, a taxa de dilatação do diâmetro dos segmentos aórticos pesquisados, foi estatisticamente maior em homens do que em mulheres (p < 0,05), com exceção daquele imediatamente proximal ao tronco celíaco (Figura 16).

**Figura 16:** Comparação da taxa de dilatação dos segmentos da aorta abdominal entre homens e mulheres nas faixas etárias de 20-30 e 61-70 anos. Em (A), no segmento proximal ao tronco celíaco; em (B), proximal à artéria renal mais alta; em (C), distal à artéria renal mais baixa; em (D), proximal à bifurcação aórtica. *: p<0,05.

## DISCUSSÃO

O envelhecimento é um processo dinâmico, progressivo e irreversível, ligado intimamente a fatores biológicos, psíquicos e sociais^5^. Seus efeitos dependem não apenas da idade, mas de sexo, estilo de vida, comorbidades, fatores socioeconômicos e influências constitucionais. Por isso, não há homogeneidade na população idosa^5^^,^^10^^,^^15^^-^17. O cirurgião deve prezar pelo conhecimento mais particularizado possível sobre as estruturas abordadas em cada procedimento, pois isso afeta positivamente a qualidade da técnica e evita lesões iatrogênicas.

A aorta e outras artérias calibrosas, principalmente as com camada elástica mais desenvolvida, tornam-se mais dilatadas, alongadas e tortuosas conforme o paciente envelhece^13^^,^^18^^,^^19^. A compreensão das modificações da anatomia arterial com o envelhecimento pode ser útil para o planejamento de procedimentos invasivos — a exemplo da opção de acesso e curvaturas de cateteres e diâmetros de balões de angioplastia e stents em um procedimento endovascular —, além de, futuramente, poder auxiliar na fabricação de materiais endovasculares com diâmetro e curvaturas que melhor se adaptem a pacientes de acordo com sexo e faixa etária.

Obviamente, quando disponíveis, o planejamento de uma intervenção deve considerar informações anatômicas fornecidas por exames não invasivos; entretanto, os resultados desta pesquisa podem ajudar a prever algumas dificuldades. Por exemplo, ao tentar um cateterismo seletivo da ARE, tendo como acesso uma punção na artéria femoral direita em um paciente de 68 anos, baseado nas diferenças de angulação encontradas no presente estudo, é provável que, em uma paciente do sexo feminino, se encontrem dificuldades maiores do que as previstas para homens da mesma faixa etária (Figura 17).

**Figura 17:** Tomografia computadorizada contrastada de abdômen em corte coronal, comparando as medidas do ângulo de origem da artéria renal esquerda (ARE) em pacientes de ambos os sexos entre 61 e 70 anos. (A) ângulo de origem da ARE em um homem; (B) ângulo de origem da ARE em uma mulher.

É interessante ressaltar que o presente estudo detectou maior tendência de dilatação arterial, como na aorta abdominal e AICD, com o avançar da idade em homens, como já sugerido por outros estudos^17^^-^22.

A AMS também apresentou maior modificação de angulação e diâmetro em homens. A menor predisposição para alterações da anatomia arterial em mulheres pode estar relacionada ao papel do estrogênio^6^^,^^9^^,^^22^, que aumenta a biodisponibilidade de óxido nítrico (NO) sem aumentar a expressão e/ou atividade da óxido nítrico-sintase endotelial (eNOS), cujas propriedades antioxidantes podem induzir ou acelerar o envelhecimento vascular^6^^,^^8^^,^^9^. A biodisponibilidade do NO é fundamental para a função endotelial normal, e sabe-se que a idade avançada leva ao comprometimento da produção endotelial de NO e ao aumento da inativação dele pelo superóxido, o que contribui para a disfunção endotelial relacionada à idade^6^^,^^10^^,^^16^^,^^23^^,^^24^.

As características mais notáveis do envelhecimento vascular descritas na literatura são as mudanças mecânicas e estruturais que ocorrem nas paredes dos vasos, dentre elas, a dilatação e o espessamento arteriais, verificados principalmente na aorta abdominal e destacados em diversas publicações^5^^,^^12^^,^^15^^,^^22^^,^^25^. As alterações arteriais ao longo da vida também são influenciadas por fatores de risco cardiovasculares modificáveis, como hipertensão, obesidade, tabagismo e estilo de vida, além de fatores não modificáveis, a exemplo de genética, idade e história familiar^15^^,^^16^^,^^21^.

Sabe-se que, mesmo em pacientes saudáveis, a rigidez aórtica acentua-se após os 50 anos de idade^7^^,^^26^, e estudos *post-mortem* mostram que o espessamento da parede aórtica durante o envelhecimento ocorre por aumento na espessura da camada íntima, mesmo em populações com baixa incidência de aterosclerose^7^^,^^8^^,^^10^^,^^15^^,^^17^. Anatomicamente, essas alterações se manifestam não apenas pelo aumento do diâmetro, mas também pelo alongamento aórtico, e, do ponto de vista fisiológico, o espessamento arterial, que se relaciona com a velocidade de onda de pulso (*pulse-wave velocity*, PWV), demonstrou ser um fator de risco independente para eventos cardiovasculares^5^^,^^9^^,^^13^^,^^14^.

Embora não tenhamos pesquisado o espessamento, as modificações de diâmetro arterial detectadas neste estudo corroboram os achados da literatura. Nossa revisão, entretanto, não encontrou estudos que correlacionassem as angulações de emergência dos ramos da aorta abdominal com o sexo e a idade dos pacientes.

Infelizmente, o caráter retrospectivo desta pesquisa permitiu acesso somente ao sexo e à faixa etária dos pacientes submetidos aos exames, o que possibilitou apenas a constatação de que o diâmetro da aorta foi maior em homens e que aumentou progressivamente com o avançar da idade em ambos os sexos (Figura 18). Diversos artigos publicados apontaram que os diâmetros da aorta são maiores em homens do que em mulheres^12^^,^^18^^-^20^,^^22^^,^^25^^,^^26^; porém, não avaliaram que a taxa de dilatação desses diâmetros é maior em homens do que em mulheres em todas as faixas etárias, como detectado no presente estudo.

**Figura 18:** Tomografia computadorizada contrastada de abdômen em corte transversal, comparando as medidas dos diâmetros da aorta abdominal proximal à artéria renal mais alta em pacientes de ambos os sexos e diferentes faixas etárias. (A) paciente do sexo masculino na faixa etária entre 20 e 30 anos; (B) paciente do sexo feminino na faixa etária entre 20 e 30 anos; (C) paciente do sexo masculino na faixa etária entre 60 e 70 anos; (D) paciente do sexo feminino na faixa etária entre 60 e 70 anos.

Assim como aconteceu com a aorta, a AICD e a AICE apresentaram diâmetro médio maior em homens do que em mulheres e, considerando-se a AICD, apresentaram aumento significativo do calibre com o passar da idade.

A literatura sobre aneurismas de artérias ilíacas é escassa, mas evidências sugerem que as ilíacas comuns não aneurismáticas têm maior calibre à direita do que à esquerda e que aneurismas, quando ocorrem, além de serem mais frequentes, tendem a apresentar maior diâmetro à direita do que à esquerda^21^^,^^27^^-^29, o que corrobora os achados de nossa casuística. Este fato pode estar relacionado à maior ocorrência de aneurismas na aorta (4 masculinos:1 feminino)^30^ e ilíacas (5 masculinos:1 feminino)^27^, já que a lei de Laplace explica que quanto maior o raio do vaso, maior a tensão exercida na parede^31^. Ou seja, por apresentarem vasos mais calibrosos, pode haver uma predisposição anatômica para homens desenvolverem aneurismas nessas artérias.

Obviamente, essa observação não explica, isoladamente, a maior ocorrência de aneurismas em segmentos de menor diâmetro, já que é conhecido que aneurismas do segmento abdominal da aorta são cerca de quatro vezes mais frequentes do que aqueles da aorta torácica, a qual apresenta maior diâmetro^13^^,^^28^. Certamente, a origem de uma doença complexa como o aneurisma é multifatorial; sabe-se, por exemplo, que há uma marcada alteração histológica da aorta ao longo da vida, já que os jovens têm fibras de elastina grossas, concêntricas e homogêneas, enquanto os idosos as apresentam mais finas e fragmentadas, ocupando menor volume na camada média da artéria^5^^,^^7^^,^^10^^,^^14^^,^^32^^,^^33^.

Para avaliar as alterações da anatomia arterial associadas à idade em pacientes de ambos os sexos, o ideal seria acompanhar com exames de imagem uma coorte de pacientes ao longo da vida. Entretanto, esse desenho de estudo apresentaria enormes empecilhos para a execução. Outras limitações desta pesquisa são número relativamente baixo de TCs e o desconhecimento de comorbidades e hábitos de vida dos pacientes, como o tabagismo, que podem predispor a alterações da anatomia arterial.

## CONCLUSÕES

O diâmetro da aorta em diversos segmentos aumenta progressivamente com o passar da idade em pacientes de ambos os sexos e, entre pacientes de mesma faixa etária, é maior em homens do que em mulheres. Os diâmetros de todos os ramos da aorta, com exceção da ARE, foram maiores em homens do que em mulheres em todas as faixas etárias. O ângulo de emergência da artéria mesentérica superior foi maior em homens do que em mulheres em todas as faixas etárias, com exceção da faixa etária entre 20 e 30 anos; o ângulo de origem da artéria renal esquerda foi maior em mulheres do que em homens com idade entre 51 e 60 anos. A taxa de dilatação dos diâmetros nos diferentes segmentos da aorta abdominal foi maior em homens do que em mulheres, com exceção do segmento proximal ao tronco celíaco.
